# Lupeol as a modulator of bacterial resistance mediated by the MepA efflux pump in *Staphylococcus aureus*

**DOI:** 10.1007/s00210-026-05342-5

**Published:** 2026-05-06

**Authors:** Nara Juliana Santos Araújo, Camila Aparecida Pereira Silva, Cicera Datiane Morais Oliveira-Tintino, Gabriel Gonçalves Alencar, Maria do Socorro Costa, Ana Raquel Pereira da Silva, Juliete Bezerra Soares, Janaína Esmeraldo Rocha, Saulo Relison Tintino, Vanessa Lima-Bezerra, Henrique Douglas Melo Coutinho, Vanessa Leopoldino Coelho Rodrigues, José Maria Barbosa-Filho, José Bezerra de Araújo-Neto, Ana Carolina Ferreira Araujo, João Arthur de Oliveira Borges, Gildênia Alves de Araújo, José Thyálisson da Costa Silva, Jacqueline Cosmo Andrade-Pinheiro

**Affiliations:** 1 Post-Graduate Program in Biochemistry and Molecular Biology, Federal University of Cariri, Barbalha, Ceará Brazil; 2 Laboratory of Applied Microbiology-LAMAP, Federal University of Cariri, Barbalha, Ceará Brazil; 3 Post-Graduate Program in Health Sciences, Federal University of Cariri, Barbalha, Ceará Brazil; 4 Federal University of Cariri, Barbalha-UFCA, Barbalha, Ceará Brazil; 5 Regional University of Cariri, Crato-URCA, Crato, Ceará Brazil; 6 Laboratory of Microbiology and Molecular Biology, Regional University of Cariri-LMBM, Crato, Ceará Brazil; 7 Federal University of Paraiba-UFPB, João Pessoa, Paraíba Brazil; 8 Post-Graduate Program in Biological Sciences, Biosciences Center, Federal University of Pernambuco., Recife, Pernambuco Brazil; 9 Technological Education Center–CET, Teresina, Piauí Brazil; 10 Post-Graduate Program in Biological Chemistry, Regional University of Cariri, Crato, Ceará Brazil

**Keywords:** Triterpenes, Molecular docking, RT-qPCR, Membrane permeability, Efflux inhibitor

## Abstract

Bacterial resistance constitutes one of the main threats to global public health, driving the search for therapeutic strategies capable of restoring the effectiveness of antimicrobials. Among the mechanisms involved, efflux pumps stand out, reducing the intracellular concentration of drugs and contributing to multidrug resistance. In this context, inhibitors of these pumps emerge as a promising approach, as they increase the intracellular concentration of antibiotics. Natural compounds, such as the triterpene lupeol, have been investigated as potential modulators of bacterial resistance due to their relevant biological activities. This study evaluated, from a mechanistic perspective, the ability of lupeol to inhibit the MepA efflux pump in the K2068 strain of *Staphylococcus aureus*. The minimum inhibitory concentration (MIC) was determined, efflux inhibition assays were performed, membrane permeability analysis was conducted using Sytox Green, ethidium bromide fluorescence was evaluated, RT-qPCR was performed, and molecular docking was performed. The results demonstrated that lupeol exhibits isolated antibacterial activity considered without clinical relevance (MIC ≥ 1024 µg/mL); however, it reduced the MIC of ciprofloxacin and ethidium bromide, indicating inhibition of the MepA pump. No alteration in membrane permeability and an increase in ethidium bromide fluorescence were observed with increasing lupeol concentration. RT-qPCR showed inhibition of *mepA* gene expression. In molecular docking, the compound presented a binding energy of − 9.01 kcal/mol, with van der Waals, hydrophobic, and hydrogen bonding interactions. It is concluded that lupeol shows potential as an efflux pump inhibitor, acting on the functional inhibition of the pump and the negative regulation of mepA gene expression, thus configuring itself as an adjuvant strategy in combating bacterial resistance.

## Introduction

Bacterial resistance to antibiotics has become one of the main threats to global public health, representing a clinical and economic challenge of great proportions (Guedes 2021; Alexandre et al. 2023). It is estimated that, by 2050, approximately 10 million people will die annually as a result of infections caused by resistant microorganisms, a phenomenon associated with increased morbidity and mortality, prolonged hospital stays, higher therapeutic costs, and limited effectiveness of available treatments (Aflakian and Hashemitabar 2025; Wesgate et al. 2020; Patini et al. 2020). This scenario is intensified by factors such as the indiscriminate use of antimicrobials, widely observed during the COVID-19 pandemic, and by genetic mechanisms that favor the maintenance and dissemination of resistance among bacterial strains (Macedo 2022).

Bacterial resistance is characterized by the ability of microorganisms to grow in the presence of antimicrobial agents that should inhibit their development (Patini et al. 2020). This phenomenon involves different strategies, such as the production of enzymes capable of degrading antibiotics, alterations in pharmacological targets, metabolic modifications, reduced influx and active elimination of drugs, and biofilm formation and horizontal gene transfer (Urban-Chmiel et al. 2022; Varela et al. 2021). Evidence suggests that such mechanisms are ancestral, possibly having arisen millions or even billions of years ago, which reinforces their evolutionary relevance (Church and McKillip, 2021).

Among these mechanisms, efflux pumps stand out as important determinants of bacterial resistance, acting as transmembrane proteins responsible for the active extrusion of toxic and antimicrobial compounds, reducing their intracellular concentration and compromising therapeutic efficacy (Cordeiro 2021; Guedes 2021; Macedo 2022; Muniz 2022; Pereira 2023). In this context, the development of efflux pump inhibitors has emerged as a promising strategy, since these compounds can block drug extrusion and restore their antimicrobial activity (Rocha 2022; Tintino et al., 2017).

Among the efflux pumps, MepA, belonging to the MATE (multidrug and toxic compound extrusion) family, stands out for its relevance in *Staphylococcus aureus*. Encoded by the *MepA* gene, part of the mepRAB operon, this protein has 451 amino acids and 12 transmembrane segments, being overexpressed in the K2068 cell line (Rocha 2022; Muniz 2022). MepA is capable of extruding different substrates, including fluoroquinolones, such as norfloxacin, as well as ethidium bromide and pentamidines, using the proton gradient associated with sodium antiport as an energy source (Rocha 2022; Muniz 2022).

In parallel, secondary metabolites of natural origin have been extensively investigated as sources of new therapeutic agents, due to their structural diversity and biological potential (Anokwah et al. 2023). Among these compounds, terpenoids stand out, exhibiting antinociceptive, antiviral, anti-inflammatory, and antibacterial properties (Silva et al. 2023). Lupeol, a pentacyclic triterpene derived from lupane, is widely distributed in fruits, vegetables, and plant species belonging to different families, including Fabaceae and Euphorbiaceae (Rend et al. 2022; AlMousa et al. 2025; Sen et al. 2024; Sharma et al. 2020; Dhanush et al. 2024; Singha et al. 2025). This compound exhibits diverse biological activities, such as antioxidant, antimicrobial, antiparasitic, anti-inflammatory, antiproliferative, and neuroprotective actions, highlighting its pharmacological potential (Das et al. 2023; Javed et al. 2021; Park et al. 2023).

Previous studies have demonstrated that Lupeol exhibits activity as an inhibitor of the NorA efflux pump in *Staphylococcus aureus*, indicating its potential as a modulator of bacterial resistance (Araújo et al. 2025). However, studies evaluating its action against other relevant efflux pumps, such as MepA, are still scarce, especially under a mechanistic approach that integrates phenotypic, molecular, and in silico analyses.

Therefore, the present study aimed to evaluate the ability of lupeol to inhibit the MepA efflux pump in the K2068 strain of *Staphylococcus aureus* to broaden the understanding of its potential as a modulator of bacterial resistance.

## Materials and methods

### Lupeol and bacterial strain

The triterpene lupeol (C₃₀H₅₀O) was acquired from Sigma-Aldrich (St. Louis, MO, USA) and solubilized in dimethyl sulfoxide (DMSO), with the final solvent concentration being less than 5% (v/v) in all assays.

The bacterial strain used was *Staphylococcus aureus* K2068, known for overexpressing the MepA efflux pump, from the microorganism collection of the Microbiology and Molecular Biology Laboratory of the Regional University of Cariri (LMBM/URCA).

### Determination of the minimum inhibitory concentration and evaluation of efflux pump modulation

The minimum inhibitory concentration (MIC) was determined by the microdilution method in 96-well plates, according to CLSI guidelines (2020). The assays were performed in triplicate, using resazurin as an indicator of bacterial viability.

The bacterial inoculum was adjusted to the 0.5 standard of the McFarland scale (≈1 × 10⁸ CFU/mL) and subsequently diluted to 1 × 10^5^ CFU/mL. The plates were incubated at 37 °C for 24 h.

After incubation, 20 µL of 0.03% (w/v) resazurin stock solution were added, and the reading was performed based on the color change indicative of bacterial growth.

The assessment of efflux activity modulation was conducted using subinhibitory concentrations (MIC/8) of lupeol and the tested compounds, to avoid a direct antibacterial effect and allow the analysis of possible modulating effects.

The plates were prepared containing bacterial inoculum, lupeol, and ciprofloxacin or ethidium bromide (EtBr) in serial dilutions (0.5–512 µg/mL) and incubated at 37 °C for 24 h (CLSI, 2020; Tintino et al. 2017).

### Membrane permeability assessment

Membrane permeability was assessed using Sytox Green dye. Suspensions of *Staphylococcus aureus* K2068, adjusted to the 0.5 standard of the McFarland scale, were distributed in 96-well black-bottom plates.

Lupeol was tested at concentrations of 50, 100, and 200 µg/mL, while polymyxin B was used as a positive control and PBS as a negative control.

After incubation for 1 h, Sytox Green (1 µM) was added, followed by an additional 30 min of incubation. Fluorescence was measured on the Cytation 1 reader (BioTek®) with Gen5™ software, using excitation wavelengths of 485 nm and emission wavelengths of 528 nm. The assays were performed in triplicate (Yuen et al. 2023).

### Fluorescence assessment of ethidium bromide

Intracellular retention of ethidium bromide (EtBr) was used as an indirect measure of efflux activity. *Staphylococcus aureus* K2068 strains were cultured and incubated at 37 °C, with the inoculum adjusted to the 0.5 standard of the McFarland scale in PBS.

The bacterial suspensions were incubated with Lupeol at concentrations of 10, 20, and 50 µg/mL. CCCP (50 µg/mL) was used as a positive control, while PBS was used as a negative control (vehicle).

After incubation for 1 h 30 min, EtBr (100 µg/mL) was added, followed by a new incubation. The samples were centrifuged, washed with PBS, and resuspended. The pellet was transferred to 96-well black-bottom plates, and fluorescence was measured on a Cytation 1 reader (BioTek®) with Gen5™ software, using excitation wavelengths of 530 nm and emission wavelengths of 590 nm. The assays were performed in triplicate (Oliveira-Tintino et al. 2023).

### Gene expression analysis by RT-qPCR

The *Staphylococcus aureus* K2068 strain was cultured in BHI medium at 37 °C for 24 h. The bacterial suspension was then incubated under two experimental conditions: (i) control, containing ciprofloxacin at a subinhibitory concentration (MIC/8) and (ii) test, containing ciprofloxacin (MIC/8) associated with Lupeol (MIC/8).

After incubation, total RNA was extracted using TRIzol™ (Thermo Fisher Scientific), preceded by cell lysis with lysozyme and Tris–EDTA buffer. The RNA was quantified and used for cDNA synthesis with the SuperScript™ VILO™ Master Mix kit (Thermo Fisher Scientific), according to the manufacturer's instructions.

The expression of the *MepA* gene was evaluated by RT-qPCR using SYBR™ Green PCR Master Mix on the qTower^3^ G instrument (Analytik Jena). The primers used are described in Table 1, with the 23S rRNA gene used as an endogenous control.

**Table 1:** Nucleotide sequence of the primers used

Gene	*Primer forward*	*Primer reverse*	Reference
*mepA*	GGCAAATAAAGGCCGTATGA	CAGTCGCTTGAAGCATACCA	(Ashraf et al. 2022)
*23 s*	ACGGAGTTACAAAGGACGAC	AGCTCAGCCTTAACGAGTAC	(Machado et al. 2018)

Relative gene expression was determined by the ΔΔCt method described by Livak and Schmittgen (2001).

### Molecular docking

The three-dimensional structures of Lupeol, ethidium bromide, ciprofloxacin, and CCCP were generated in MarvInSketch software (ChemAxon Ltd., version 23.14) and optimized with Open Babel (version 2.4.1), using the MMFF94 force field.

The three-dimensional structure of the MepA protein was modeled by homology on the SWISS-MODEL server, using the UniProt Q2G140 sequence from *Staphylococcus aureus* (NCTC 8325/PS 47) and the structural model available in the PDB (Robin et al. 2024).

Molecular docking was performed in AutoDock Vina (version 1.2.5), with the aid of AutoDock Tools (MGLTools, version 1.5.7), using a 50 Å grid box centered on the conveyor cavity (*x* = − 32.554; *y* = 0.012; *z* = 11.605).

The docking validation followed the methodology described by Martin et al. (2024), considering a root-mean-square deviation (RMSD) of less than 2.0 Å (Trott and Olson 2010; Martin et al. 2024).

The results were expressed in binding energy (kcal/mol), with more negative values indicating greater affinity.

### Statistical analyses

Statistical analysis was performed using GraphPad Prism software. Comparisons between groups were conducted using analysis of variance (ANOVA), followed by appropriate post-hoc tests. Results were expressed as mean ± standard error of the mean, with *p*-values < 0.05 considered statistically significant.

## Results

### Determination of the minimum inhibitory concentration and evaluation of efflux pump modulation

After determining the minimum inhibitory concentration (MIC), it was observed that lupeol did not show relevant antibacterial activity when evaluated in isolation, with MIC ≥ 1024 µg/mL, which characterized the absence of a significant antibacterial effect under the tested conditions.

**Fig. 1:**
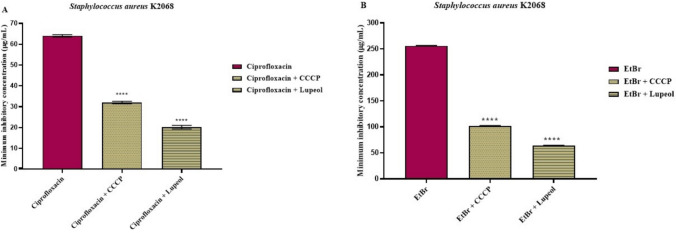
Modulation of efflux pump activity by Lupeol in *Staphylococcus aureus* K2068, assessed by changes in the minimum inhibitory concentration (MIC). (A) Ciprofloxacin and (B) ethidium bromide in the presence or absence of Lupeol and CCCP. The reduction in MIC values indicates increased intracellular accumulation of the compounds, suggesting inhibition of efflux activity. Statistical analysis performed by one-way ANOVA, followed by Bonferroni post hoc test. ^****^p < 0.0001 compared to the control

In the evaluation of efflux activity modulation (Fig. 1), the association of lupeol with ciprofloxacin (1a) promotes a reduction in the MIC from 64 (control) to 20.2 µg/mL. In the association of ciprofloxacin with the positive control CCCP, the MIC is reduced to 32 µg/mL.

Similarly, in the presence of ethidium bromide (1b), the MIC was reduced from 256 (control) to 64 µg/mL with the use of Lupeol, while CCCP promoted a reduction to 101.2 µg/mL.

The reduction in the MIC of known substrates of the MepA efflux pump in the presence of lupeol suggests a modulating effect on this system, favoring the intracellular accumulation of the tested compounds. These findings indicate that lupeol may act as a modulator of efflux activity, contributing to the restoration of bacterial susceptibility to the evaluated agents.

### Membrane permeability assessment and fluorescence assessment of ethidium bromide (EtBr)

In the evaluation of membrane permeability by Sytox Green, it was observed that none of the tested lupeol concentrations promoted a significant change in fluorescence intensity compared to the negative control, indicating no effect on bacterial membrane integrity.

**Fig. 2:**
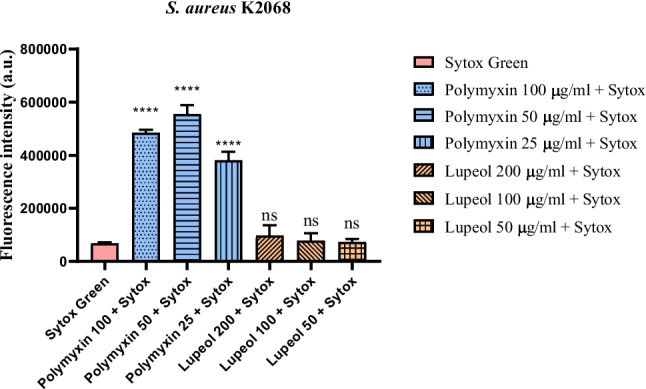
Evaluation of membrane permeability in *Staphylococcus aureus* K2068 using the SYTOX Green assay. Fluorescence intensity (a.u.) reflects membrane damage, with increased fluorescence indicating compromised cell integrity. Lupeol did not promote a significant change compared to the negative control (PBS), while polymyxin B (positive control) increased fluorescence levels. Statistical analysis was performed using one-way ANOVA, followed by Tukey’s test. ^****^p < 0.0001 vs. control; ns = not significant

The positive control (polymyxin B) shows increased fluorescence, confirming the sensitivity of the assay (Fig. 2). The variation observed between different concentrations of the positive control may be associated with experimental effects, such as dye saturation or concentration-dependent interactions. Although the results indicate preservation of membrane integrity, complementary analyses, such as microscopy, may strengthen this observation in future studies.

**Fig. 3:**
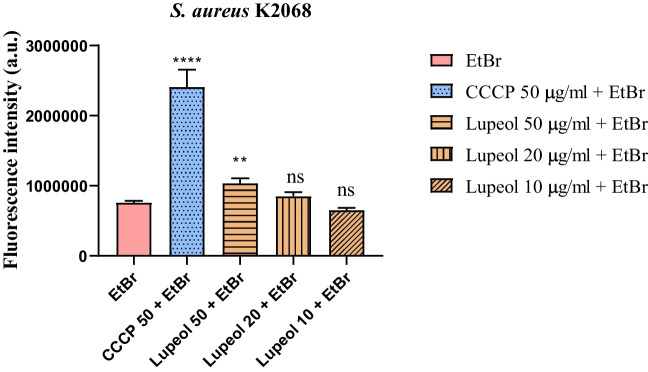
Evaluation of intracellular retention of ethidium bromide (EtBr) in *Staphylococcus aureus* K2068 treated with lupeol. Fluorescence intensity (a.u.) reflects the intracellular accumulation of EtBr, with increased fluorescence indicating reduced efflux activity. Lupeol promoted a moderate increase in fluorescence, more evident at a concentration of 50 µg/mL, while CCCP (positive control) showed a more pronounced effect. Statistical analysis was performed using one-way ANOVA, followed by Tukey’s test. ^****^p < 0.0001; ^**^p < 0.01; ns = not significant

In the evaluation of intracellular retention of ethidium bromide, an increase in fluorescence was observed in cells treated with lupeol, more evident at a concentration of 50 µg/mL. This increase did not show a clearly concentration-dependent pattern, being characterized as moderate when compared to the CCCP positive control, which promotes greater dye retention (Fig. 3). These data suggest a possible effect of lupeol on modulating the activity of the MepA efflux pump.

### Gene expression analysis by RT-qPCR

Gene expression analysis demonstrated that the combination of ciprofloxacin with lupeol resulted in a significant reduction in *MepA* gene expression compared to treatment with ciprofloxacin alone.

**Fig. 4:**
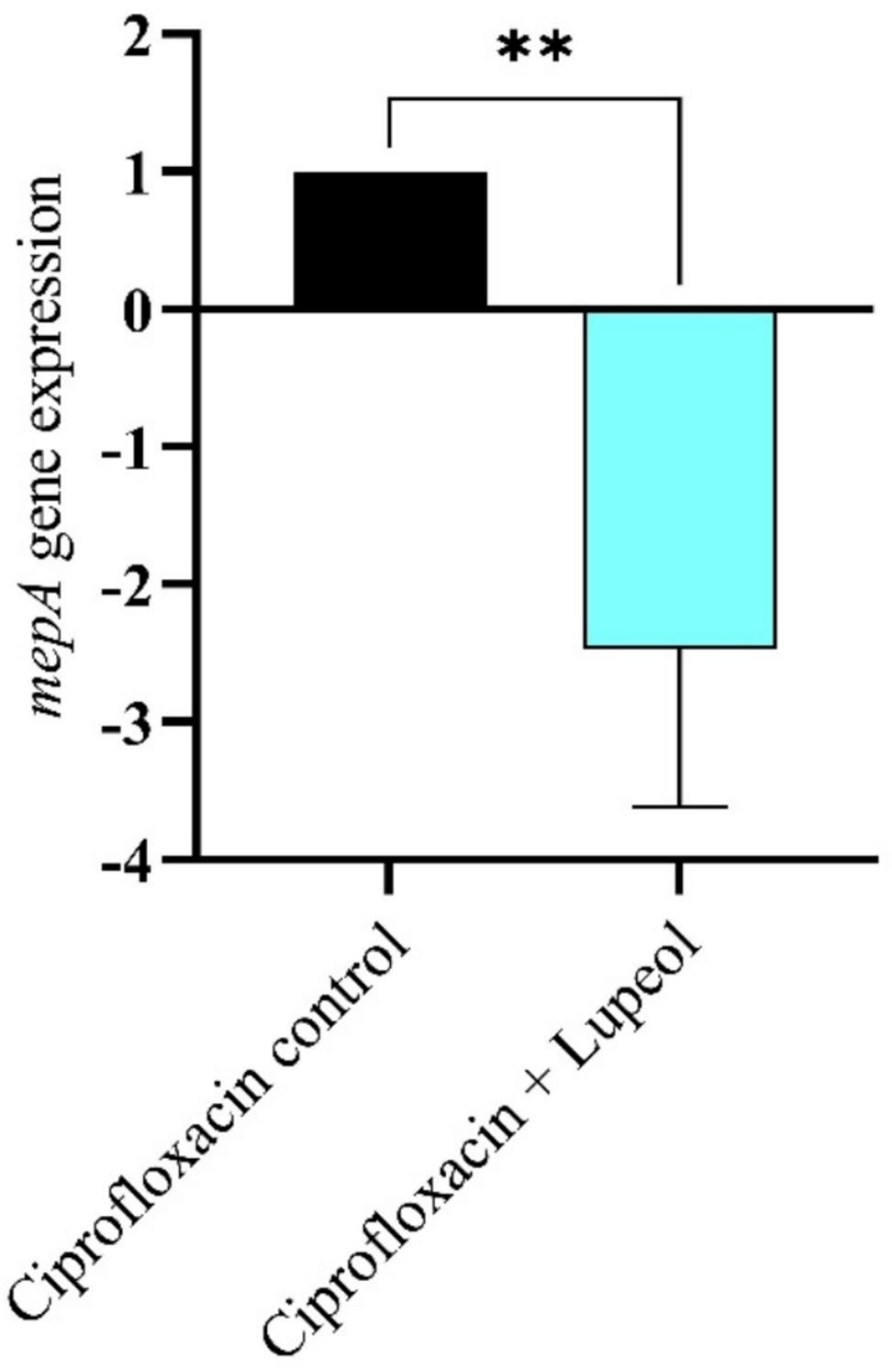
Relative expression of the mepA gene in *Staphylococcus aureus* K2068 determined by RT-qPCR using the 2^-ΔΔCt method. The comparison was made between ciprofloxacin alone and ciprofloxacin combined with Lupeol. The values represent the fold change in expression compared to the control group. Statistical analysis was performed using Student’s *t*-test. ^**^p < 0.01 indicates a significant difference between the groups

The ΔCt value ≈ − 2.5 corresponds to an approximate 5.7-fold decrease in gene expression, indicating a modulating effect of Lupeol on the bacterial efflux system (Fig. 4).

It should be noted that the effect of lupeol was evaluated exclusively in combination with ciprofloxacin under the experimental conditions employed.

### Molecular docking

Molecular docking of Lupeol with the MepA protein model revealed a binding energy of − 9.01 kcal/mol, indicating a high affinity between the compound and the protein binding site.

**Fig. 5:**
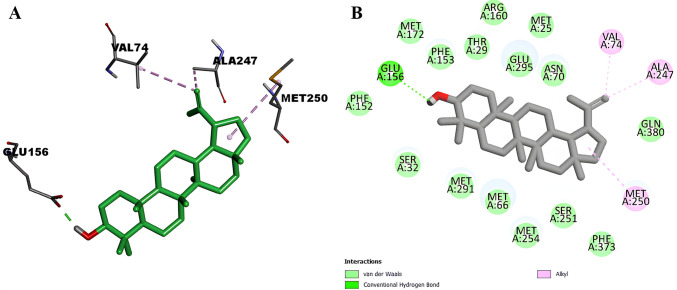
Molecular docking analysis of lupeol interaction with the MepA efflux pump. (A) Lupeol binding conformation at the protein’s active site. (B) Representation of molecular interactions, including hydrogen bonds and hydrophobic interactions with amino acid residues

Analysis of the interactions demonstrated the formation of a hydrogen bond with the Glu156 residue (1.90 Å), in addition to hydrophobic interactions with Ala247 (3.47 Å), Val74 (4.17 Å), and Met250 (4.86 Å). Van der Waals interactions are also identified with different residues, including methionine, phenylalanine, and serine (Fig. 5).

In conjunction with the data present in Table 2, it can be observed that lupeol has a relevant affinity for the MepA transporter, suggesting potential interaction with regions important for its function, which corroborates the experimental results obtained in the biological assays.

**Table 2:** Docking results with ciprofloxacin, ethidium bromide, and CCCP

Results	Ciprofloxacin	Ethidium bromide	CCCP
Binding energy	− 8.236 kcal/mol	− 8.596 kcal/mol	− 7.493 kcal/mol
Conventional hydrogen bond	Ala247 (1.70) Gln380 (2.32) Glu156 (2.12)	Ala247 (2.51) Gln157 (2.71) Glu295 (2.50) Ser251 (2.78)	Arg160 (2.33) Asn70 (3.00) Gln157 (2.49)
Carbon–hydrogen bond	Glu295 (3.28)	––	––
Pi–anion interaction	Glu295 (4.01)	Arg160 (3.76) Glu295 (3.89)	Glu295 (4.24)
Alkyl interaction	Val74 (4.21) Val298 (5.21)	Val74 (4.47)	Val298 (3.93)
Pi–alkyl interaction	Ala247 (5.05) Val74 (5.11)	Val74 (5.40)	Ala247 (5.11)
Pi–sigma interaction	––	––	Val74 (3.44)
Pi–sulfur interaction	––	Met250 (5.02)	––

^*^Distances (in parentheses) are expressed in Angstroms (Å)

## Discussion

Analysis of the results demonstrated that lupeol does not exhibit significant intrinsic antibacterial activity (CLSI, 2024; Kadeřábková et al. 2024) when evaluated in isolation (MIC ≥ 1024 µg/mL), indicating low efficacy as a direct antimicrobial agent under the tested conditions. However, the compound was able to reduce the MIC of substrates such as ciprofloxacin and ethidium bromide, suggesting a modulating effect on the activity of the MepA efflux pump.

Previous studies report that Lupeol may exhibit antibacterial activity against different microorganisms, including Gram-positive and Gram-negative bacteria (Rosandy et al. 2021). However, the antimicrobial activity of triterpenoids is not uniform, often being influenced by factors such as chemical structure, bioavailability, and mechanisms of bacterial resistance (Al-Ansi et al. 2024; Piddock 2006; Li; Nikaido 2003).

In this context, the results of the present study are in agreement with evidence indicating that these compounds may have low efficacy as direct antimicrobial agents, but act as modulators of bacterial resistance.

The positive control CCCP acts as an uncoupler of the proton gradient, dissipating energy and indirectly inhibiting the activity of the efflux pump through changes in the electrochemical potential of the membrane (Tintino, 2017). In contrast, lupeol did not show an effect on cell membrane permeability, suggesting a distinct mechanism, possibly more specific and unrelated to membrane destabilization, which may represent a pharmacological advantage, as its structure favors interaction with specific lipid domains, affecting the fluidity and functional activity of transmembrane proteins, including active transporters, such as MATE pumps (Nonato et al. 2021; Souza 2023).

The ethidium bromide fluorescence emission assay demonstrated the presence of a moderate increase in intracellular fluorescence, more evident at higher concentrations of lupeol, indicating a partial reduction in efflux activity. The occurrence of this effect, even if lower than that presented by CCCP, corroborates the hypothesis of functional modulation of the MepA pump, since the increase in intracellular retention of ethidium bromide is directly associated with the inhibition of bacterial efflux systems in fluorometric assays (Ahmad et al., 2024; Whittle et al. 2019).

In addition, RT-qPCR analysis showed a reduction in *MepA* gene expression in the presence of lupeol, suggesting that the compound may act not only on the functional activity of the pump, but also at the transcriptional level. These findings broaden the understanding of lupeol’s potential as a modulator of bacterial resistance, since the overexpression of efflux pump genes is directly associated with the resistance phenotype and its modulation can result in a reduction in the MIC of antimicrobials (Long et al.; 2024; Meng et al. 2025).

Molecular docking results reinforce this hypothesis by demonstrating a favorable interaction between lupeol and the MepA protein, including hydrophobic interactions and hydrogen bonding with residues relevant to the transporter’s function, such as Glu156, previously described as essential for the pump’s activity (Sapula and Brown, 2016). Hydrophobic interactions between substrates and the MepA pump have already been reported in the literature, reinforcing the relevance of hydrophobic domains in the structure of MATE family efflux pumps (Silva et al. 2022; Almeida et al. 2020). This interaction pattern is compatible with the functional model of MepA, whose architecture includes binding sites rich in nonpolar residues, fundamental for the recognition and translocation of lipophilic compounds.

Although previous studies by the group have demonstrated the potential of lupeol as an inhibitor of the NorA efflux pump (Araújo et al. 2025), the present work expands this knowledge by investigating its action on the MepA pump, belonging to the MATE family, suggesting that the compound may present a broader spectrum of modulation of efflux systems.

As a limitation, the use of only one clinical strain stands out, which may restrict the generalization of the results. Therefore, additional studies using different bacterial strains and complementary experimental approaches are needed to confirm and expand the findings presented.

## Conclusions

This research proposes an exploratory approach to investigating isolated lupeol as a potential modulator of bacterial resistance. The results obtained highlight its ability to inhibit the MepA efflux pump, a central mechanism in bacterial resistance. Although the minimum inhibitory concentration of lupeol did not show clinical relevance, the data relating to membrane permeability and fluorescence with ethidium bromide suggest an association with the modulation of efflux systems, unrelated to direct interactions on the cell wall. In addition, RT-qPCR analysis indicated a reduction in the expression of the *MepA* gene, reinforcing the compound’s action also at the transcriptional level.

The molecular docking results indicate a robust interaction between lupeol and the target protein, with a binding energy of − 9.01 kcal/mol. The conformation of lupeol, involving hydrogen bonds and hydrophobic interactions with specific residues, reinforces its potential as a valuable tool in the modulation of efflux pumps.

These findings highlight the need for further investigations, both to optimize the structure of lupeol in pursuit of greater efficacy and to expand understanding of its potential therapeutic implications.

## Data Availability

The data supporting the conclusions of this study are available upon reasonable request to the corresponding author.
